# DISPEL: A Python Framework for Developing Measures From Digital Health Technologies

**DOI:** 10.1109/OJEMB.2024.3402531

**Published:** 2024-05-17

**Authors:** A. Scotland, G. Cosne, A. Juraver, A. Karatsidis, J. Penalver-Andres, E. Bartholomé, C. M. Kanzler, C. Mazzà, D. Roggen, C. Hinchliffe, S. Del Din, S. Belachew

**Affiliations:** Biogen, Cambridge, Massachusetts 2191 Cambridge MA 02142 USA; Translational and Clinical Research Institute, The Catalyst 151515 NE4 5TG Newcastle upon Tyne U.K.

**Keywords:** Signal processing, digital biomarkers, digital health technology, python, gait, balance, cognition, drawing, smartphone, wearable sensing, inertial sensor

## Abstract

*Goal*: This paper introduces DISPEL, a Python framework to facilitate development of sensor-derived measures (SDMs) from data collected with digital health technologies in the context of therapeutic development for neurodegenerative diseases. *Methods*: Modularity, integrability and flexibility were achieved adopting an object-oriented architecture for data modelling and SDM extraction, which also allowed standardizing SDM generation, naming, storage, and documentation. Additionally, a functionality was designed to implement systematic flagging of missing data and unexpected user behaviors, both frequent in unsupervised monitoring. *Results*: DISPEL is available under MIT license. It already supports formats from different data providers and allows traceable end-to-end processing from raw data collected with wearables and smartphones to structured SDM datasets. Novel and literature-based signal processing approaches currently allow to extract SDMs from 16 structured tests (including six questionnaires), assessing overall disability and quality of life, and measuring performance outcomes of cognition, manual dexterity, and mobility. *Conclusion*: DISPEL supports SDM development for clinical trials by providing a production-grade Python framework and a large set of already implemented SDMs. While the framework has already been refined based on clinical trials’ data, ad-hoc validation of the provided algorithms in their specific context of use is recommended to the users.

## I. Introduction

Reducing duration, size and costs of clinical trials is a compelling need in the development of therapeutic solutions for neurodegenerative diseases. Digital health technologies (DHTs) and sensor-derived measures (SDMs) of motor and cognitive function are promising solutions to this problem. Their development and validation lifecycle, however, is complex and it might take years before adequate technology readiness levels are reached through cross-sectional and longitudinal randomized control trials [1], [2], [3]. Tools to shorten this cycle are hence highly needed, including suitable computational frameworks for SDM extraction. Available open-source libraries for the development of SDMs, in fact, are often limited to very specific applications or device sensor locations [4], [5], [6]. Even recent efforts towards a code library for integration of diverse biomarkers [7] or for effective standardization [8], [9] do not seem to have yet achieved the needed level of traceability and generalizability.

This paper presents a Python framework, named DISPEL, designed to facilitate development and validation of SDMs within the context of therapeutic development applied to neurodegenerative diseases. Specifically, to facilitate the fulfilment of evolving, and potentially uncharted, regulatory requirements for DHTs and for digital biomarker and endpoint development and acceptance [3], the following objectives were identified: a) allow to ingest data collected with different DHTs and experimental protocols; b) offer a traceable process for the extraction of SDMs from raw data; c) manage the complexity associated to the data that are typically recorded in clinical trials and real world scenarios; d) flagging unexpected user-behaviors potentially affecting the SDM values; e) enable community contributions in the form of data formats and signal processing modules for SDMs extraction.

## II. Materials and Methods

DISPEL is made of building blocks developed according to good software development practices such as test-driven development and peer-review. These blocks served as a foundation for development and enabled to turn prototype algorithms into production-grade software adhering to good software development practices. Robust testing was achieved by regression testing of SDMs, peer-review and, where feasible, enhancing unit tests with synthetic data or controlled experiments with expected outcomes.

DISPEL was designed to support custom and third-party data formats. Ad-hoc *input output (io)* modules and guidance for new *io* implementations facilitate reading from different sources. This is expected to encourage integrating new data types and/or code modules, irrespective of device type and location.

Modularity, integrability and flexibility were adopted to enable processing of data from structured performance outcome assessments/tests (e.g., six-minute walk, finger drawing, etc.). These tests can entail different experimental protocols, typically composed of sub-tasks, which in DISPEL are structured in so-called *levels*. For example, if, in a smartphone-based drawing task, the subject is asked to draw four different shapes twice with both hands, each attempt is a *level* and the shape type and hand side are *modalities*. This data modelling approach allows for high flexibility and simplifies coding when processing multiple raw data sets and extracting multiple SDMs at a time. Moreover, it enables extracting SDMs only for specific tasks and sub-tasks and processing only specific *levels*, optimizing computational resources and management of incomplete datasets.

Transparency was ensured by adopting an object-oriented design, where each *DataSet* and *MeasureSet* object contains attributes indicating source and definition (e.g., units), while each processing step class is linked to its input and output data.

The object-oriented design also enabled standardization in SDMs computation, naming and storing. Automatically generated SDM names directly link to the referring task, level, and modality, while automatically generated documentation summarizes specific computation settings. Information needed to build data trace graphs is generated for all transformation and extraction steps.

Measure extraction functionalities are organized in modules per test and measures are wrapped in a class (measure collection) to simplify handling multiple data collections and aggregations (e.g., multiple measures from same tests, average over different levels, etc.). Wide usability of the library was pursued by enabling measure collections to be exported into a Python dictionary, a JSON file, or a CSV file. Quality monitoring is facilitated by flags, which can be linked during the processing steps to specific measures, levels or readings. These flags are automatically stored in the data model and allow to track technical (e.g., unstable sampling frequency) or user-related factors (e.g., wrong handling of a smartphone).

## III. Results

**Fig. 1.**
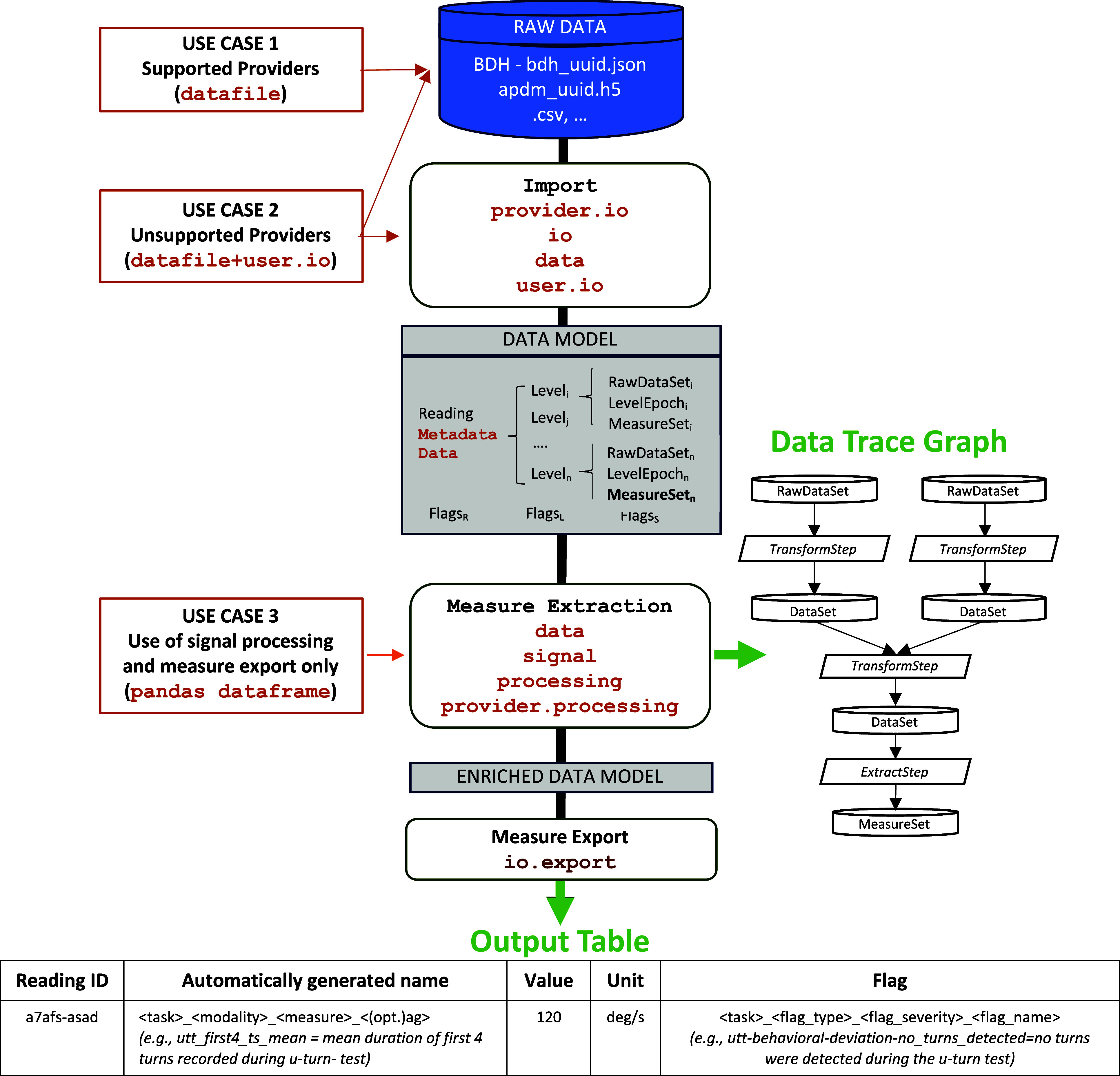
Figure shows the core functionalities of DISPEL (white blocks) and the modules implementing them (orange text). The orange rectangular boxes represent the different scenarios in which a user could interact with DISPEL and the type of data and modules they would need to implement and provide as an input in each case. The grey box in the middle of the chart details the content of the data model. This consists of a reading (including data and metadata) structured in a series of levels (Level_i_) that may contain different sensor data (RawDataSet), custom-defined windows of analysis (LevelEpoch) and calculated measures (MeasureSet). The Model also includes the flags generated if technical issues or user-related deviations in the test executions are detected. Exerts of info for a data trace graph and SDMs tables obtained as an output are also illustrated, together with an example of automatically standardized measures and flags naming.

DISPEL is freely accessible at https://github.com/newcastleuniversity/DISPEL/. Fig. 1 summarizes the implementation of the various modules and the library outputs, together with use cases representing encouraged contributions from the DHT community.

DISPEL ingests data from multiple providers. Originally designed to process data from three mobile data collection applications (Ad Scientiam, Biogen Digital Health Konectom, and SensorLog - .json), it currently also reads data from a wearable sensor provider (APDM Wearable Technologies - .h5) and in the Mobilise-D format [10].

Implemented algorithms, partly taken from the literature and partly newly developed, extract SDMs from 16 structured wearable- or smartphone-based tests (including 6 questionnaires), assessing overall disability and quality of life, and measuring performance outcomes in the domains of cognition, manual dexterity and mobility (see Table I, exemplifying also currently flagged user behaviors).

**TABLE I:** Tests, Modalities, User-Related Flags and Measures

Domain	Tests	Input data	Sub-Tasks (Modalities)	User-related flags	Example measures
**Quality of life & disability**	Mood	Activity sequence (questions, timestamps, responses)	-	-	Median Total Score
	Physical state				Minimum value for score
	MSIS-29				Spasms in limbs
	ALSFRS				Total Score
	PDQ-39				Mobility Score
	Neuro-QOL				Anxiety t-Score
**Cognition**	Cognitive Processing Speed	Acceleration, Angular Velocity, Touch Events, Activity Sequence	Digit-to-digit (Random/Pre-defined)	Phone not held in portrait mode	Mean reaction time (predefined sequence, random key, symbol 9)
			Symbol-to-digit (Random/Pre-defined)		
			Key (Random/Fixed)		
**Upper limb function**	Pinch	Acceleration, Angular Velocity, Touch Events	Bubble sizes (Small/Medium/Large Extra-Large)	No recorded pinch attempt	Mean accuracy normalized by duration (square clockwise, right hand, first attempt)
			Hand (Left/Right)		
	Drawing	Acceleration, Angular Velocity, Touch Events	Shape (Circle/Spiral/Square)	Phone not held in portrait mode	Mean accuracy normalized by duration (square clockwise, right hand, first attempt)
			Direction (Clockwise/Counter-clockwise)		
			Hand (Left/Right)		
	Grip Force	Pressure	Hand (Left/Right)	-	Median reaction time (left)
	Finger Tapping	Acceleration, Angular Velocity, Touch Events	Hand (Left/Right)	Simultaneous tapping with both fingers	Total number of valid taps
	Typing	Key Typed, Word Sequence, Touch Events	Correctness (Correct/Incorrect)	Usage of autocomplete	Standard deviation time interval (correct)
	Pronation Supination	Acceleration, Angular Velocity	Hand (Left/Right)	Rotation range smaller than expected	Median movement amplitude (left)
**Mobility**	5 U-Turn	Acceleration, Angular Velocity, Magnetometer	Static Balance	Excessive motion detected	95% Ellipse area after 5s adjustment
			U-Turn (Number of turns)	Not enough turns performed	Mean absolute turn speed (first 4 turns)
	Walking	Acceleration, Angular Velocity, Magnetometer, GPS (relative)	Six-minutes walking (Continuous, Breaks, Turns)	Too many breaks and turns	Mean walking speed (excluding periods with GPS displacement <1m)
			Two- minutes walking (2 Minutes, 6 Minutes)	Phone not in expected position	Gait variability (first two minutes)
	Gait continous monitoring	Acceleration, Angular Velocity, Magnetometer	-	-	Minutes of activity recorded during the day

Overview of structured tests, data quality flags associated with user errors in test execution, and example measures that can be currently extracted with DISPEL from wearables or smartphones. In the upper limb function tests the smartphone is expected to be handheld, while in the mobility tests it is expected to be worn on the back using a running belt. Equivalent locations are expected for alternative wearables when relevant. MSIS-29: Multiple Sclerosis Impact Scale. ALSFRS: Amyotrophic Lateral Sclerosis Functional Rating Scale. PDQ-39: Parkinson's Disease Questionnaire. QOL: Quality of Life.

## IV. Discussion

This paper aimed to provide the DHT community with a tool to accelerate the development of SDMs for clinical trials. To this end, DISPEL offers a production-grade Python framework, which already extracts a large set of SDMs from sixteen assessments/tests.

Compared to other available tools, one of DISPEL's strengths is its generalizability to different providers and DHTs. This is also accompanied by a unique way of simultaneously warranting high transparency, modularity and flexibility. DISPEL is also novel in the way it leverages the advantages of object-oriented codebase to deal with the complexity of data from real-life unsupervised settings. In fact, its modular and customizable pipeline of processing steps allow for programming simultaneous SDM computations and handling of problematic steps (e.g., data missingness or signal corruption). Furthermore, it includes an innovative solution for flagging technical and user-related factors affecting SDMs values. All these attributes of DISPEL are essential when operating in the highly regulated intended use of drug development clinical trials. DISPEL is also expected to facilitate reproducibility and transferability of results across the community.

DISPEL includes both published and unpublished measures extraction algorithms. While these have been extensively tested and optimized for end-use based on data from clinical trials in neurodegenerative disorders [11], [12], [13], their analytical and clinical validation [1], [2], [3] should be the focus of further dedicated efforts.

## V. Conclusion

DISPEL offers a Python framework to generate SDMs from various DHT data providers and is readily extendable to incorporate additional input and processing modules. We hope to see DISPEL becoming a widely adopted and community-driven framework, to accelerate digital biomarker/endpoint development and to support collaborative efforts between industry, academia, and regulators to drive DHT literacy and adoption.

## Author Contributions Statement

AS conceived the original design of DISPEL and coordinated its development. AS, GC, AJ, AK, and JPA implemented the library and the documentation of the code. CH and SDD supported the integration of additional I/O modules and contributed to the library documentation. CMK, CM, DR, EB, and SB supervised the scientific developments related to the definition of sensor derived measures of mobility, dexterity, and cognition. CM produced the initial draft of the manuscript. GC and JPA designed the figures and tables. All co-authors critically reviewed the initial draft and contributed to the development of the final manuscript.

## Conflict of Interest Statement

AS, GC, AJ, AK, JPA, EB, CMK, CM, DR, and SB were employees of Biogen at the time of developing this paper and might hold shares of the company. SDD reports consultancy activity with Hoffmann-La Roche Ltd. outside of this study.
